# An examination of the factors fueling migration amongst Community Service practitioners

**DOI:** 10.4102/phcfm.v6i1.625

**Published:** 2014-11-07

**Authors:** Candice Reardon, Gavin George

**Affiliations:** 1 Health Economics and HIV and AIDS Research Division (HEARD), University of KwaZulu-Natal, South Africa

## Abstract

**Background:**

Research is needed in order to understand the potential influence of the Bilateral Agreement between South Africa and the United Kingdom (UK), as well as other more recent international and local policies restricting movement of South African health workers abroad; and to determine what effect they have on the migration intentions and plans of health professionals in South Africa.

**Aim:**

The aims were to (1) explore the migration intentions and the factors that influence these intentions amongst Community Service (CS) nurses and doctors; (2) explore their views and opinions about the Bilateral Agreement between the UK and South Africa (SA) and other UK policies around the recruitment and employment of foreign health professionals; and (3) understand the impact of these policies on the migration plans of these CS doctors and nurses.

**Method:**

Qualitative focus groups and interviews were conducted with 23 CS doctors and nurses. To supplement this, 6 interviews were conducted with nurses and a doctor who had worked in the UK.

**Results:**

A higher disposition toward moving abroad was apparent amongst those who had experienced a challenging and frustrating CS year. Poor working conditions, including long work hours, high patient loads and inadequate resources and equipment, as well as low salaries and the perceived ambivalence of the government to the complaints of health practitioners, were influencing decisions to migrate abroad.

**Conclusion:**

The findings suggest that government efforts to better manage, recognise and respect the work and contribution of health professionals to the country would go a long way toward retaining health professionals.

## Abstrait

**Un examen des facteurs d'incitation à la migration des professionnels de la santé et de leurs opinions sur la politique restreignant leurs déplacements**.

**Contexte:**

Un accord bilatéral et d'autres politiques existent aujourd'hui entre le Royaume-Uni et l'Afrique du Sud, visant à endiguer la migration des professionnels de la santé en Afrique du Sud vers le Royaume-Uni. Des recherches sont nécessaires pour comprendre l'influence potentielle de cet accord bilatéral, et d'autres politiques interdisant la circulation des travailleurs de la santé sud-africains à l’étranger, et l'effet qu'ils ont sur les intentions d’émigration et les plans des professionnels de la santé en Afrique du Sud.

**Objectifs:**

Les objectifs étaient spécifiquement (1) d'explorer les intentions d’émigration des infirmières et des médecins des services communautaires, ainsi que les facteurs qui influent sur ces intentions, (2) d'explorer leurs points de vue et opinions à propos de l'accord bilatéral entre le Royaume-Uni et SA et à propos des autres politiques du Royaume-Uni sur le recrutement et l'emploi des professionnels de santé étrangers, et (3) de comprendre l'impact de ces politiques sur les plans d’émigration des médecins et des infirmières de services communautaires.

**Méthodes:**

Des groupes de discussion et des entrevues qualitatives ont été organisés avec 23 médecins et infirmières de services communautaires. Nous avons eu six entretiens avec des infirmières et un médecin qui avaient travaillé au Royaume-Uni.

**Résultats:**

Une plus grande disposition à se déplacer à l’étranger était évidente parmi ceux qui avaient connu une année de service communautaire difficile et frustrante. Les mauvaises conditions de travail, y compris de longues heures de travail, les charges élevées de patients et l'insuffisance des ressources et de l’équipement ainsi que les bas salaires et l'ambivalence apparente du gouvernement aux plaintes des praticiens de la santé ont influencé ces médecins à émigrer à l’étranger.

**Conclusions:**

Les résultats suggèrent que les efforts du gouvernement pour améliorer la gestion, reconnaître et respecter le travail et la contribution des professionnels de la santé dans le pays contribuerait grandement à retenir les professionnels de la santé.

## Introduction

The health workforce constitutes one of the 6 key building blocks for a well-functioning health system.^1^ In 2006, the World Health Organization (WHO) estimated that 37% of trained doctors and 7% of trained nurses had migrated from South Africa (SA) to work abroad.^2^ The emigration rate of nurses is calculated to be between 1% and 2% per year, which means that by 2008, between 2128 and 4256 nurses would have left the country.^3^ By 2015, it has been estimated that SA will experience a critical ‘gap’ of 15 380 staff nurses, 22 121 professional nurses, 3930 doctors and 5677 medical specialists.^4^

Evidence suggests that health professionals are considering emigration as a result of the prospects of a better life in their destination country.^5^ Crush, Pendleton and Tevera,^5^ in their 2005 study, found that a substantial proportion of South African health professionals had already taken active steps to emigrate. Similarly, in 2007, Pendleton, Crush and Lefko-Everett^6^ found that 13.9% of the registered South African health professionals in their study had applied for work permits, 5.5% were in the process of applying, 6.2% had applied for or were applying for a permanent residence permit in their destination country and roughly 30.2% had already applied for professional registration in their country of interest. South African health professionals showed a preference for destination countries such as Australia and New Zealand, the United Kingdom (UK), Europe, North America and Canada.^6^

Other studies amongst South African heath workers revealed that financial factors, better job opportunities for themselves and schooling opportunities for children abroad as well as the high crime rate in SA were significant factors encouraging these health workers to emigrate.^7^ Another important pull factor attracting health professionals to countries overseas is the opportunity to gain international experience.^8^ According to Oberoi and Lin,^9^ however, push factors motivating health professionals to leave SA can exert a more powerful influence on their emigration decisions than the factors attracting them to their destination country. The major push factors that emerged most frequently in their interviews were poor remuneration, lack of job satisfaction, lack of future prospects for further education and career development, poor working conditions, HIV and poor quality of life.^9^ In a South African study, medical and nursing students cited poor working conditions within the public health sector, including long work hours, high patient loads, inadequate resources and occupational hazards as being the primary push factors encouraging them to consider leaving the country.^10^

In SA, there have been various initiatives by the government specifically aimed at strengthening the rural healthcare system that has been weakened by internal migration to the private health sector and the migration of health workers to other countries. The development and implementation of the Occupation Specific Dispensation (OSD) was based on the recognition that improvement in the conditions of service and remuneration for health professionals constituted the most urgent priority. Dispensations for specific occupational categories include unique salary structures for each identified occupation in the public service, centrally-determined grades and job profiles, career-progression opportunities and other employment practices.^11^

The introduction of Community Service (CS) for all categories of health professionals was aimed at further alleviating staff shortages in rural and under-served areas. It has been argued that the introduction of CS has not stopped doctors from leaving to spend time overseas after their CS year. Reid^12^ found that an increasing proportion of CS doctors between 1999 (34%) and 2001 (43%) intended to work overseas after their CS year. Of Reid's sample, 20% said they would consider working in rural or under-served areas in the future, which he argues is adequate reason for the government to implement an appropriate and systemic set of incentives that could encourage these health professionals to practise in rural areas, circumventing the requirement for young health professionals to endure their year of obligatory service.^12^ Based on these findings it has been argued that CS defeats its own end because health professionals may assume they have fulfilled their duty and compensated society for the costs of their study.^12, 13^ Other opponents have thrown harsher criticism at the current policy around medical internships and CS, referring to it as ‘cruel and degrading’ with ‘no other professional group in the country subjected to such levels of exploitation and discrimination by the state’.^13^ A lack of supervision, compounded by extraordinary high levels of overtime, have been identified as two key problems of CS requiring urgent attention.^14^

SA has entered into a number of bilateral agreements with foreign governments aimed at moderating the outflow of South African health professionals and alleviating health worker shortages in the country. In the 1990s, the South African government began placing pressure on the UK to refrain from recruiting South African doctors. In 1999, the former President of SA, Nelson Mandela, publicly accused the UK of poaching South Africa's health professionals.^15^ This culminated in the endorsement of a Memorandum of Understanding (MOU)^16^ between the UK and SA in 2002 as a response to the recruitment of health workers from developing countries. The MOU, although no longer in force today, took a dynamic flexible approach to encouraging ongoing dialogue between the UK and SA around human resources for health, healthcare capacity and development with regular assessment and adjustment by both countries.^17^ The Bilateral Agreement covered a wide range of areas with a view to sharing and disseminating best practice between the two countries. It also offered the opportunity for nationals from both the UK and SA to benefit from time-limited placements in either country, thereby creating transcultural opportunities for both clinical personnel and healthcare managers.^8, 15^

Around the same time as the Bilateral Agreement between SA and the UK was in existence, the UK *Code of Practice for the International Recruitment of Healthcare Professionals*,^18^ developed in 2001, prohibited National Health Service organisations from recruiting health workers from certain countries, including SA, unless a government-to-government agreement existed with that country. Although this was respected widely by the public sector, private-sector organisations sustained their recruitment efforts, resulting in a net rise in foreign health workers in the UK in 2002.^19^ Consequently, the UK Code of Practice was revised in 2004 to ensure that all healthcare organisations, including the private sector, adhered to its recruitment guidelines as well as to cover the recruitment of both temporary and permanent staff.^18^

### Objective of the study

There is a paucity of empirical research studies that have examined the views and opinions of CS practitioners toward the Bilateral Agreement between the UK and SA^16^ and other policies such as the UK Code of Practice,^18^ which sought to curb the large outflow of South African health workers to the UK. One of the research objectives of this study was to understand how the increased difficulty faced by South African health professionals in gaining employment in the UK and other countries, may influence their future emigration plans and intentions. Moreover, this study sought to gain an understanding of the emigration considerations and intentions of CS doctors and nurses, including both the push and pull factors that influence their decisions to remain in or leave SA.

To provide a point of comparison with the data from the CS practitioners, interviews were conducted with South African nurses and doctors that had worked in the UK in order to establish the factors that fuelled their migration abroad. The attitudes and opinions of the returned health professionals toward the Bilateral Agreement and stricter UK policies were also sought in order to provide an important point of comparison between older health professionals who have worked abroad and younger nurses and doctors who have not.

### Contribution to the field

Nurses and doctors coming to the end of their obligatory CS are at a critical stage in their careers. There is research to suggest that fewer CS practitioners are wanting to remain in the public service after their year of CS^12^ and that the proportion wanting to go overseas after their year of obligatory service has increased.^20^ In view of this, this research sought to explore the work experiences of health professionals during their CS year and any influence this may have on their intentions and plans to leave SA. Moreover, this study showcases the main factors fuelling their migration desires and intentions, as well as how these may influence their migration plans in view of stricter policies around immigration and employment in the UK.

## Research method and design

### Design

A qualitative approach was used for this study in order to provide rich and in-depth explorations and verbal descriptions which resulted in sufficient detail to describe the views and opinions of CS practitioners toward migration and foreign employment policies.

### Sampling strategy

The researchers were granted access to 2 public hospitals in KwaZulu-Natal. In total, 29 participants took part in this study. This figure comprised 23 CS nurses and doctors (15 CS doctors and 8 CS nurses) and 6 health professionals (5 nurses and 1 doctor) who had worked in the UK. Twenty-one participants were women and 8 were men. Twenty participants were black South Africans, 7 were Indian and 2 were white South Africans. The focus groups and interviews were all conducted in early 2011.

In order to access CS nurses and doctors, two hospitals were approached for the study. Approval was sought from the medical managers at both hospitals to interview their CS nurses and doctors. The researcher was unable to contact the nursing manager in order to set up focus groups with the CS nurses at the Durban hospital. Altogether, two focus groups with CS nurses and two with CS doctors were conducted at a district hospital located in the Pietermaritzburg area and four focus groups with CS doctors were carried out at a regional-level hospital located in the greater Durban area. All CS doctors and nurses in the two hospitals were notified in writing about the study by their CS representative or their medical or nursing manager and a proposed schedule of focus group dates and times circulated to them. The researchers had originally intended to conduct eight focus groups because of the study's time constraints, thus only eight focus group sessions were planned for. The CS practitioners were under no obligation to attend the focus group sessions; those interested self-selected into the study.

To supplement this data, six interviews were conducted with nurses and doctors who had worked abroad in the UK. These individuals were sourced from the database of Africa Health Placements (AHP), a social profit organisation who had helped find them employment on their return from the UK. Two interviews with a doctor and nurse who had worked in the UK were conducted over the phone. The other four interviews were conducted in person with the nurses at their hospitals.

### Materials

The focus group discussion questions were developed in alignment with the research objectives. The overarching categories of questions included: (1) their emigration considerations and reasons for emigrating or wanting to emigrate; (2) personal views and opinions about the Bilateral Agreement and other UK policies; and (3) the potential impact of these policies on their emigration plans and intentions. The interview schedule used with the nurses and doctor who had worked in the UK sought to solicit their views and opinions about the Bilateral Agreement between SA and the UK as well as other UK immigration and employment policies, their reasons for wanting to work abroad and their comparative experiences of working in the UK and SA.

### Procedure

The focus groups and interviews were conducted at a time that was convenient for the nurses and doctors at their place of employment and lasted between 40 and 50 minutes. Two interviews with a doctor and nurse who had worked in the UK were conducted over the phone. The other four interviews were conducted in person with the nurses at their hospitals. The fieldwork was conducted by a female, junior researcher with no medical training. The focus groups and interviews were recorded and transcribed verbatim.

### Analysis

As recommended by Bogdan and Biklen,^21^ the transcripts were read twice by the researcher in order to familiarise herself with the data before coding began. The coding was data driven as themes were allowed to emerge from the text. Opler's terminology of ‘expressions’ and ‘themes’ was used in coding the data.^22^ ‘Expressions’ denote the basic units of data or incidents in the text that develop into themes and subthemes through a process of synthesising and comparison across cases.

Descriptive codes were used to identify basic expressions that were found in individual transcripts. These were compared with expressions emerging from other interviews and, after being categorised into subthemes, were given more analytical coding labels. Ryan and Bernard's^22^ techniques for identifying themes from basic expressions in the text were used. The process of analysing the data moved from descriptive to analytical coding as recommended by Gibbs.^23^

### Ethical considerations

Ethical approval was gained from the University of KwaZulu-Natal (UKZN) Biomedical Research Ethics Committee (Ref: BE191/010) as well as from the KwaZulu-Natal Provincial Department of Health (Ref: HRKM146/10). All participants involved in the study were informed about the nature of the study and what their participation would entail. Ethical issues such as the voluntary and informed nature of participation, anonymity and confidentiality were addressed within the informed consent forms that were handed to the respondents. The focus groups and interviews were conducted in private rooms at the hospitals, at a time that was convenient for the participants. The interviews and focus groups were recorded with the respondents’ permission. All identifying information has been taken off the transcripts so as to ensure confidentiality of the respondents’ accounts. Every participant was provided with a copy of the final report after completion of the study, as prescribed by good ethical practice.^23^ In the presentation of the findings, no identifying information of the participants or the hospitals is provided in order to ensure confidentiality of their accounts.

## Results

### Migration considerations

All but four doctors were considering the possibility of emigrating at some stage in their careers. The desire to work abroad for a temporary period of time before they decide on permanent settlement abroad was reported often. The doctors who were less inclined to want to leave the country were located in one hospital in the Pietermaritzburg area and reported having a positive CS year. In comparison, the group of doctors who voiced the strongest desire to leave the country were from the hospital situated in the greater Durban area; these doctors expressed many frustrations and complaints about their CS experience.

Most of the doctors considering emigration shared the same plans; they would specialise in SA and after that reassess the situation in the country and their job prospects, only emigrating if they thought it necessary.

In contrast, the majority of the nurses had not given much thought to leaving SA and did not have much knowledge or information about working and living abroad. Some nurses (*n* = 4) expressed an openness to the possibility of emigrating, although this was not articulated with any definite plans to do so. A number of significant deterrents to migrating abroad emerged from the nurses’ group discussions that appeared to root them in the country for at least the near future. These included the contractual obligation they had with the provincial Department of Health, caring for their children and the lower-job grade that they would probably work at overseas. In particular, the nurses expressed dislike for the work that some expatriate nurses abroad had to endure in nursing homes, which included caring for the elderly and looking after their less ‘medical’ needs. The following quote from a community service nurse indicates that she regards the nature of the work done in care homes as degrading and undignified for themselves as professional nurses, which might dissuade her from working abroad:

‘She was a sister when she was in here in SA and then when she went there she was making tea for the *gogos* there [*Gogos* *is a Zulu word commonly used in SA to refer to elderly women or grandmothers*]. Why you making tea for those? So there it's just bringing your dignity down.’ (Community service nurse, Regional public hospital in Pietermaritzburg area)

### Push and pull factors for working abroad

### Push factors

It was agreed unanimously that push factors were stronger motivations weighing in on participants’ emigration considerations than pull factors. The influence that push factors play in the nurses and doctors’ desire to migrate abroad is reflected in the following quote by a doctor:

‘There's no reason why we should want to leave … Like I said, I love South Africa and I will always regard this as home … However there are factors here that make me think twice about remaining. And it's definitely the push factors because even money-wise you can make a living in South Africa … it's just there's too many push factors that force you out of here.’ (CS doctor, Regional public hospital in greater Durban area)

These push factors included poor salaries, poor and frustrating working conditions, job uncertainty after CS, crime and the poor state of the healthcare system in SA. One doctor shared frustrations about not being able to afford his basic material needs even after qualifying as a doctor:

‘I've been driving [*a*] Jetta 2 1990 model, after Medical School, after graduating … So frustrating … Go overseas, make quick bucks and come back, get the car and all those things. So we can't even buy – I'm talking about a car here … we can't even afford to buy a house, after years and years of graduating from Medical School. Those things are so frustrating and pushing you to go.’ (CS doctor, Regional public hospital in greater Durban area)

The increase in salaries brought about by the OSD was viewed positively by some respondents from both hospitals, whilst others felt it would not have the desired effect of attracting and retaining health workers in the public healthcare system. One doctor from the Pietermaritzburg hospital believed that the OSD would obviate the problem of medical doctors having to work abroad after qualifying to pay off study loans. Another CS doctor, who had worked previously in the UK, recognised the salary benefit from the OSD and believed it was far more lucrative to work in the public rather than the private health sector. However, two doctors in the same focus group disagreed with this doctor's sentiments about the OSD. They believed that the salary increases from the OSD were not strong enough incentives to deter them and other doctors from going overseas. Their beliefs about the inadequacy of the OSD are reflected in the following quote:

‘I mean it's quite dependent on your overtime … you should be able to get a salary, a decent salary where you were able to pay for your house and your car and educate your children and all of that, without having to do overtime. But if you look at it, we're not getting paid enough. I mean if you don't do overtime then it's quite a big chunk of your salary … It's not a good enough incentive, I think, to stay.’ (CS doctor, Regional public hospital in greater Durban area)

Secondly, several doctors complained about experiencing very poor working conditions that were making them debate whether to remain in the country. Their primary complaints centred on a lack of proper medical equipment and a high burden of disease and patient mortality. Several doctors expressed sadness and frustration about not being able to do more for their patients because of the lack of resources and heavy patient loads, which they felt limited their ability to deliver good patient care.

Other factors related to the healthcare system, such as a lack of money being invested for improvements, the poor treatment of doctors and the severe health worker shortages, were also identified as being significant push factors. Severe staff shortages could leave doctors feeling as though they were constantly being forced to ‘fill the gap’. Another doctor reported that the shortages in her hospital had made her stagnate in her medical training because of the lack of senior staff available for supervision and mentoring. She believed that the UK would offer her a better-resourced setting with better learning opportunities.

Finally, several doctors shared their anxieties and concerns about their job opportunities following their CS year. One doctor lamented that he was ‘currently facing unemployment’ as a result of the freezing of posts which he blamed on the government's frugal investment of money into the healthcare system. Although two doctors, both based in the Pietermaritzburg hospital, were against their fellow doctors leaving SA, they expressed understanding and sympathy for their fellow white South African doctors who, they believed, were inclined to pursue work opportunities abroad because of the lack of specialist training posts available to them. The issue of particular race groups being at a disadvantage in the selection for specialist posts was also articulated by three doctors in the Durban hospital who believed that they would receive fairer opportunities for enrolment in specialist training programmes in the UK or Canada. In the following quote, one doctor describes her experience of being turned down for a position in a specialist training programme in the province:

‘I've applied for a registrar post and I got a very nice reply from the head of the department stating that the fact that 67% of the province of KZN is made up of black South Africans, therefore they have to give 67% of their posts to black South Africans; so therefore you have a better chance of applying to one of the other Med Schools in the country. So I feel lately I would stand a better chance there [*in the UK*], not being discriminated by the colour of your skin because I think I'm a good doctor and you should be given a fair opportunity.’ (CS doctor, Hospital in greater Durban area)

#### Pull factors

The most common pull factors reported included the higher salaries and earning potential in foreign countries, better training and job opportunities, opportunities to travel and practise first-world medicine, as well as having a better quality of life. Several nurses, including those having worked in the UK, reported that higher salaries abroad, which would enable them to fulfill their children's educational and material needs, were a key motivator to migrate. In the following quote, a nurse articulates how the higher salary she received in the UK helped their family's financial situation:

‘The only thing that pushed me out of the country was that my girls were all going to the University. Three of them, one after the other, so I couldn't finance them to that extent, so I had to leave the country just to try and make some money to pay for them.’ (Nurse, District public hospital, Rural northern KZN)

Conversely, family relationships and commitments played an important role insofar as encouraging three doctors, all from the Pietermaritzburg hospital, to remain in the country to work. However, these doctors were referring to the importance of sustaining the relationships with their siblings and parents and did not have children to consider.

The improved quality of life abroad included perceptions of a safer environment for themselves and their family, reasonable working hours and patient loads as well as a higher standard of living. For one doctor, central to the better working environment overseas was the lower levels of stress he would endure and the increased resources that would reduce his ‘suffering’ at work:

‘The two main benefits – basically financially [sic] and working in a stress free environment. You know, not suffering, calling the nurse for this and that and trying to find this, trying to find needles and you can't find them, you prescribe a drug for a patient and it's not there or unaffordable … and the workload … I mean people that have worked there say that it's just a stress-free environment.’ (CS doctor, Regional public hospital in greater Durban area)

The ability to travel more easily if one were to be based in the UK or Canada was also appealing to many of the doctors. Several doctors also mentioned wanting to experience practising medicine in a first-world context, where there were fewer resource shortages and more manageable patient loads. It was also believed that working abroad would provide better opportunities to really help the patients they treated as well as to develop as a doctor.

Three of the nurses who had worked in the UK alluded to a curiosity that attracted them to working abroad. Coupled with their curiosity about working in a foreign country was their desire to gain experience working as a nurse or doctor in a foreign healthcare system that would provide them with valuable experience that they could re-invest into SA's healthcare system on their return. Fewer contractual constraints and the flexibility to work where you want and when you want, as opposed to the restrictions against moonlighting for health professionals in SA, were attractive features of work life abroad for a few of the CS nurses interviewed.

### Views about stricter UK immigration and employment policies

The doctors who were reluctant to leave SA were more supportive of the Bilateral Agreement and the stricter policy changes in the UK. Three doctors believed that the impact of the stricter policies and the ban on active recruitment from developing nations such as SA would curb the huge outflow of South African doctors and improve the healthcare system. In the following quote, one doctor emphasises the overall benefit to the South African health system and the country as a whole that stricter migration policies can produce:

‘I think it's maybe a good thing because it obviously keeps more doctors in the country; the hospitals become better staffed so the patient care gets better. So I think for the country it's actually a better thing. Probably for the doctors themselves it's not very helpful, but for the country, I think it's actually better.’ (CS doctor, District public hospital in Pietermaritzburg area)

The stricter policies around the employment and emigration of South African doctors to the UK, as well as the difficulty this created for doctors wanting to leave, also garnered support from three doctors on the grounds that it would ensure that the doctors who are leaving are serious about going to work abroad. It was felt that the ban on active recruitment was a good thing because the ease of gaining access to the UK through an agency would encourage more doctors to go abroad, including those who were less committed and resolved in their motivation to work there. One doctor also felt that the introduction of the Professional and Linguistic Assessments Board (PLAB) exams for doctors wanting to work in the UK was a good policy change; firstly, because it would ensure that only high quality doctors from South Africa were entering the UK workforce and secondly, because the exams will help prepare South African doctors for the working environment in the UK. The PLAB exam is the main route by which International Medical Graduates demonstrate that they have the necessary skills and knowledge to practise medicine in the UK.

The majority of nurses seemed either indifferent to the policies or thought they were ‘fair’. It is reasoned that the indifference expressed by some of the nurses occurred because many of them were not entertaining definite plans to go abroad and, thus, these policies were irrelevant for them in their prospective career path at the time of the study.

The majority of the doctors, however, shared negative views and frustrations relating to the stricter policies on immigration, employment and recruitment of South African doctors in the UK. In most instances, the South African government was the target of their frustration and complaints, rather than the UK government who had implemented the UK Code of Practice.^18^ The negative views expressed by the doctors related to three subthemes: (1) the restriction to their freedom to emigrate; (2) being discriminated against because of their occupation; and (3) the South African government's unfair treatment of them.

Several doctors and two nurses argued that the stricter policies and the prohibition against recruiting South African doctors to the UK constituted a restriction to their freedom to migrate. There was a shared belief that health professionals should be free to decide which country they want to work in and for how long. Owing to the greater difficulty for doctors to enter the UK, five doctors described feeling ‘forced’ to remain in country, yet in earlier discussions many of these same doctors reported feeling ‘pushed out’ or ‘forced to leave’ the country because of their negative working conditions. This predicament – being pushed to go even though they did not want to, but forced to stay – is expressed by a doctor in the following statement:

‘They've taken the short cut blocking the border saying “you can't get out”. You have to stay whether you like it or not and the conditions are not going to improve, in fact they are going to get worse. After this they increase the internship from one year to two years and the CS... they know they've blocked the borders, we can't do anything. You have to stay you have to do all those things.’ (CS doctor, Regional public hospital in greater Durban area)

Many doctors complained that the policies were unfair because they discriminated against them as health professionals. Furthermore, it was agreed widely that the South African government was unfair in its attempt to prohibit the active recruitment of South African health professionals by foreign governments. The perceived unfairness also related to their failure to address the poor working conditions causing the doctors to want to migrate and not treating them as well as health professionals are treated by foreign governments. The South African government was seen to be taking ‘short cuts’ and acting ‘selfishly’ and ‘in their own interests’. Rather than working toward prohibiting active recruitment of health professionals, the doctors reported that the South African government should take action to encourage health professionals to remain in the country:

‘You see, the government and the UK has [*sic*] a bilateral agreement – doctors are not involved. You see our department, they just make these laws and say “this is what you have to do”, without coming to us and finding out the reasons why people are moving. Instead of looking at those reasons and trying to address them, they take short cuts in trying to prevent doctors going … that's why we say if we get the opportunity we're heading out.’ (CS doctor, Regional public hospital in greater Durban area)

The relative ease with which UK doctors could come to SA without having to write the PLAB exam was a source of frustration and fuelled perception about unfair treatment by both governments. Two doctors, who intended to remain in SA, also felt that the UK doctors coming to SA should write a similar entrance exam to the PLAB exams in the UK to ensure that no preference was seen to be given to foreign doctors from developed nations.

### Potential impact of the Bilateral Agreement and stricter policies on emigration

Participants were asked what kind of impact they felt the stricter UK policies and the prohibition of recruitment of South African doctors by the UK would have on doctors’ emigration plans. The doctors believed that the policies could have three different levels of impact: (1) they would reduce the outflow of doctors to the UK; (2) they would encourage doctors migrate to other countries; or (3) they would not deter those health workers with strong motivations to migrate.

Only three doctors believed that the stricter policies would assist in reducing the number of doctors migrating abroad. Several doctors believed that South African doctors will simply try to work in another country to which they are able to gain easier entry. The ease with which South African doctors can gain entry into other first-world countries such as Canada would reportedly influence two of the doctors to emigrate to Canada rather than the UK, even though the UK was the preferred country destination. In the following statement, one doctor explains how difficulties in gaining entry into the UK will not deter her from leaving SA:

‘I'm looking at emigration out of South Africa … If Ireland won't take me I will look for somewhere else who is willing to take me if the environment is better for my lifestyle, for my family, that type of thing. I'm not going to stay in South Africa just because they've restricted me from going to the UK.’ (CS doctor, Regional public hospital in greater Durban area)

Several doctors considering migrating to the UK in the future said that the stricter policies and the difficulty in gaining access to the country would not deter them or other healthcare professionals from moving to the UK if they felt the long-term benefit to their lives and careers was worth the difficulty in getting there. Even doctors intending to remain in SA agreed that the stricter policies and difficult immigration processes would not deter those doctors who were fed up with enduring their frustrations in SA.

## Discussion

The findings indicate that the overwhelming majority of doctors have a desire to work abroad after their CS year, with some intending to specialise in SA before they move abroad and others considering migration should they not find a medical officer post after their CS year. This concurs with other quantitative studies^5, 6, 10^ which have found that a large proportion of medical and nursing students in SA are considering migrating abroad within a few years after their graduation.

Our findings also confirm those of Oberoi and Lin,^9^ in that push factors appeared to exert a stronger influence on participants’ desires and decisions to emigrate than pull factors. The most salient push factors articulated by the doctors and nurses included poor salaries, poor working conditions and resource shortages, heavy workloads, job uncertainty after their CS and the overall poor state and lack of investment in the healthcare system. Family relationships constituted either a push or pull factor, depending on the nature of the relationship; CS nurses with children were more inclined to see working abroad as an opportunity to provide better for their children, whilst CS doctors’ relationships with their parents, siblings and extended families encouraged them to remain in the country.

The most predominant pull factors included higher salaries and earning potential in foreign countries, better training and job opportunities, opportunities to travel and practise first-world medicine, as well as the possibility of a better quality of life. The allure of better salaries abroad is a common finding in the literature.^9, 10, 19, 24^ Amongst nurses, better salary prospects abroad were highly sought after in the context of their children's educational and material needs, as were the perceived flexibility and autonomy of being able to work where and when they wanted as opposed to their current contractual constraints and the restrictions against moonlighting in other health facilities. Perceptions of an improved quality of life included a less stressful and frustrating work environment as well as prospects of a higher standard of living and a safer environment within which to live.

The nature of participants’ views, whether favourable or unfavourable, about the stricter policies for recruitment and employment of foreign health professionals in the UK and the Bilateral Agreement, appeared to be associated with their migration desires and current level of job satisfaction. There were mixed views as to whether the introduction of the OSD has had or will have a potential impact on the exodus of healthcare professionals from SA; and again, these views seemed to be informed by participants’ migration desires and intentions. In contrast to some doctors’ disbelief that the OSD could curb emigration, research has shown that the OSD has made significant strides in closing the gap between the salaries earned abroad and those now earned in SA.^25^

Views supporting stricter policies restricting the migration of SA health workers abroad were premised on the perceived overall benefit that would accrue to the health system and the country should more doctors be available in the public sector. Furthermore, it was believed that stricter policies would ensure that only the most resolved health professionals would succeed. Most views and opinions expressed regarding the Bilateral Agreement and the stricter migration and recruitment policies tended to be negative, however, evoking frustration and anger amongst participants. Negative views were often directed at the South African government and were perceived as a restriction to their freedom, discrimination against them as health professionals and unfair treatment. The Melbourne Manifesto (A Code of Practice for the International Recruitment of Health Care Professionals),^26^ adopted in 2002, places a degree of responsibility on countries who are experiencing substantial losses of health professionals to examine the reasons for these outflows, particularly with regard to addressing working conditions, incentives and educational opportunities, in order to encourage health professionals to remain in the country.^26^

The majority of participants, particularly those with a desire and intention to migrate, believed that the policies would not deter doctors, including themselves, from attempting to pursue work opportunities in the UK and would possibly even result in health professionals trying to enter other first-world countries in order to escape the frustrating and challenging work environment of the public health system in SA.

### Limitations of the study

The groups of CS practitioners were not representative of the population of CS nurses and doctors in the province because of the way in which the participants were accessed. The focus groups had to be arranged at a time that was convenient for most of the doctors and nurses, which excluded several of the doctors and nurses as a result of their working schedules. Because of this, the CS nurses and doctors self-selected for the study, which could bring about differences between the sample selected for the study and the CS nurses and doctors who did not take part. Furthermore, the fact that the focus groups with the CS doctors were conducted in the last month of their CS year may have influenced their thoughts around migrating and their anxieties and concerns around the perceived job uncertainty for them in the following year.

### Recommendations

The findings underscore the importance of ensuring that CS nurses and doctors experience a fulfilling and productive CS year in order to help retain them in the public sector (and the country) thereafter. The findings also underscore the need for the South African government to have a better engagement with and inform health professionals about policies and bilateral agreements that can directly affect their work experiences and migration plans. In view of the findings, it is believed that this would go a long way toward improving health professionals’ perceptions of the government as their employer, as well as toward placating their frustrations regarding working in the public healthcare system. The government has acknowledged the importance of better management of its health workforce through garnering a deeper understanding of the key issues and challenges facing its workforce.^4^ Our findings reinforce these efforts to strengthen Human Resource Management strategies, suggesting greater attention be paid to salient push factors identified by this study as well as to better engagement with regard to migration policies, bilateral agreements and even temporary migration opportunities available to health professionals.

If the South African government intends to embark on efforts to encourage the return of South African health professionals working abroad, it is then imperative that it begins to improve the working conditions and general state of the public healthcare system in South Africa which may have incited these health professionals to leave in the first place. This is in line with recommendations made by the Code of Practice for the International Recruitment of Health Care Professionals (Melbourne Manifesto).^26^ Although salary increases have been achieved through the implementation of the OSD, the findings of this study suggest that increased salaries without improvements to working conditions and resources will not be sufficient to encourage health professionals to return to or remain in SA.

## Conclusion

The majority of CS doctors in our study expressed desires and intentions to migrate abroad shortly after completion of their CS. The push factors, relating to the conditions in which they work, their salaries and the state of the healthcare system, had a strong influence on their decision to leave the country. During this critical early period in the careers of nurses and doctors, the findings show that it is of utmost importance that these practitioners be made to feel that they have been engaged with in an adequate fashion by the South African government with regard to their complaints about their working conditions, low salaries and heavy workloads. For many considering migrating, the knowledge that the government would attempt to or was in the process of improving their working conditions would make them reconsider leaving. In this way, government efforts to better manage, recognise and respect the work and contribution of health professionals to the country may go a long way toward retaining its health professionals. Related to this was a need for the government to inform its health professionals more effectively regarding the relevant policies and bilateral agreements that have a direct effect on their practice. This could prevent the problem of health professionals misconstruing government's actions, which was found to further erode the relationship between the government and its health professionals.
